# Impact of the coronavirus disease 2019 (COVID-19) pandemic on tumor stage progression in urological malignancies: a comparative study

**DOI:** 10.3389/fruro.2025.1619185

**Published:** 2025-08-06

**Authors:** Alper Keskin, Enis Mert Yorulmaz, Kursad Donmez, Serkan Ozcan, Osman Kose, Sacit Nuri Gorgel, Yigit Akin

**Affiliations:** ^1^ Department of Urology, Denizli State Hospital, Denizli, Türkiye; ^2^ Department of Urology, Izmir Katip Celebi University, Izmir, Türkiye; ^3^ Department of Urology, Izmir Katip Celebi University Ataturk Training and Research Hospital, Izmir, Türkiye

**Keywords:** COVID-19, pandemic delay, tumor progression, urological cancers, pathological stage

## Abstract

**Objective:**

To determine whether delays in care during the coronavirus pandemic 2019 (COVID-19) were associated with pathological stage progression in urological malignancies by comparing surgical outcomes between pre-pandemic era (PREP) and pandemic-era (POSTP) cohorts.

**Methods:**

We conducted a retrospective before-and-after cohort study at a tertiary academic center. A total of 368 patients underwent radical surgeries for prostate (n=176), bladder (n=78), kidney (n=78), or testicular (n=36) cancers between April 2019 and March 2022. Patients were grouped into PREP (April 2019–March 2020) and POSTP (April 2020–March 2022) cohorts. Clinical, laboratory, and pathological data were compared using Student’s t-test, Mann–Whitney U test, Chi-square test, or Fisher’s exact test, with p<0.05 considered statistically significant.

**Results:**

POSTP prostatectomy patients had significantly higher preoperative PSA levels (13.2 ± 16.2 vs. 7.7 ± 4.5 ng/mL, p<0.001), greater tumor involvement (17.0% vs. 11.5%, p=0.019), and increased extraprostatic extension (33.7% vs. 11.9%, p=0.006) compared to PREP patients. Renal tumors were significantly larger during the pandemic (7.4 cm vs. 6.0 cm, p=0.01), and preoperative hemoglobin levels were lower (11.7 vs. 12.9 g/dL, p<0.001), suggesting more advanced disease. No statistically significant differences were observed in pathological staging for bladder or testicular cancers between the two periods (all p>0.05).

**Conclusion:**

COVID-19-related care disruptions were associated with adverse pathological features in prostate and renal cancers. In contrast, bladder and testicular cancers showed no significant stage migration. These findings emphasize the need for resilient cancer care pathways to prevent progression during future healthcare crises.

## Introduction

Urological malignancies—including prostate, bladder, kidney, and testicular cancers—are among the most common solid tumors globally and represent a substantial burden in terms of both incidence and mortality. According to recent statistics, prostate cancer (PCa) remains the second most frequently diagnosed cancer in men worldwide, with increasing incidence trends in many regions (1) Metin girmek için buraya tıklayın veya dokunun. Bladder and kidney cancers rank among the top ten most common cancers globally, while testicular cancer, though rare, is the most prevalent malignancy in younger males and requires prompt management to preserve high cure rates (2, 3).

Early detection and timely surgical treatment are key determinants of oncologic outcomes in urological cancers. However, the COVID-19 pandemic, first declared by the World Health Organization in March 2020, created an unprecedented disruption in global healthcare systems. Many countries, including Turkey, suspended elective procedures—including cancer surgeries—in late March 2020 to redirect resources toward pandemic management (4). This shift led to delays in diagnosis, staging, and definitive surgical interventions for a wide range of malignancies, raising concerns about disease progression and stage migration.

Several modeling studies predicted that delays in oncologic diagnosis and treatment during the pandemic would lead to an increase in cancer-related mortality and more advanced disease presentations. In the United Kingdom, Maringe et al. projected thousands of excess cancer deaths due to diagnostic delays across multiple tumor types (5), while Sud et al. reported that even modest disruptions in referral pathways could significantly impact survival in several cancers (6). More recently, Barclay et al. conducted a nationwide cohort analysis and confirmed that the pandemic led to significant shifts in cancer incidence and short-term survival outcomes for common tumors (7).

Despite this growing body of evidence, there is a relative paucity of data specifically evaluating the impact of the pandemic on urologic oncology. Urological malignancies encompass a wide spectrum of biological behaviors and urgency: low-risk PCa may be amenable to short-term surveillance, whereas high-grade bladder cancers (BCa) or testicular cancers (TCa) can progress rapidly in the absence of timely intervention (8). Understanding how pandemic-era cohort disruptions influenced these diverse tumor types remains a critical knowledge gap.

Additionally, researchers have speculated that COVID-19 may have biological interactions with certain urologic cancers. For instance, Bahmad et al. hypothesized a potential role for androgen-regulated pathways—particularly in PCa—in modulating COVID-19 severity or outcomes (9). While such interactions remain theoretical, the more immediate concern lies in the structural disruptions of cancer care delivery during the pandemic.

Therefore, in this retrospective cohort study, we aimed to systematically evaluate the impact of the COVID-19 pandemic on pathological stage and other prognostic indicators in patients undergoing radical surgery for prostate, bladder, kidney, and testicular cancers. By comparing pre-pandemic (PREP) (April 2019–March 2020) and pandemic-era (POSTP) (April 2020–March 2022) cohorts, we sought to determine whether care delays were associated with tumor stage progression. These findings aim to guide future healthcare preparedness strategies during global crises that may similarly affect timely cancer diagnosis and treatment.

## Materials and methods

### Study design

This study was designed as a retrospective before-and-after cohort study to evaluate potential differences in pathological tumor stage and other prognostic indicators among patients who underwent radical uro-oncological surgeries during the COVID-19 pandemic. The study compared two time-defined cohorts based on the timing of surgery: the PREP period (April 2019 – March 2020) and the POSTP period (April 2020 – March 2022). April 2020 was chosen as the cutoff because elective surgeries, including oncological procedures, were officially suspended across the country in late March 2020 due to the surge in COVID-19 cases and the reallocation of hospital resources.

### Patient selection

Patients who underwent radical prostatectomy (RP), radical cystectomy (RC), radical nephrectomy (RN), or radical orchiectomy (RO) at the Urology Department of Izmir Katip Celebi University Ataturk Training and Research Hospital during the study period were retrospectively reviewed. Only patients with pathologically confirmed uro-oncological malignancies were included. Patients were excluded if they (i) had benign pathology on final histopathology, (ii) underwent emergent surgeries unrelated to cancer, or (iii) had missing or incomplete clinical or pathological data.

### Ethics approval

The study protocol was approved by the Ethics Committee of Izmir Katip Celebi University (Decision number: 0286, dated June 16, 2022).

### Data collection

Demographic, clinical, and pathological data were extracted from electronic hospital records. Collected variables included patient age, sex, comorbidities (e.g., hypertension, diabetes mellitus), presenting symptoms, preoperative laboratory values (e.g., PSA, hemoglobin, AFP), imaging findings, tumor characteristics (size, histology, stage), and surgical outcomes. Histopathological staging and grading were conducted according to contemporary guideline-based criteria for each malignancy.

### Statistical analysis

Statistical analyses were performed using SPSS for Windows version 17.0. Continuous variables were expressed as mean ± standard deviation or median (interquartile range) depending on distribution, and categorical variables as frequencies and percentages. The Kolmogorov–Smirnov and Shapiro–Wilk tests were used to assess normality. Group comparisons for normally distributed continuous variables were conducted using Student’s t-test, while the Mann–Whitney U test was applied for non-normally distributed data. Categorical variables were compared using Chi-square or Fisher’s exact test as appropriate. A p-value <0.05 was considered statistically significant.

Only univariate analyses were conducted in this study. Although multivariable logistic regression models were considered to assess independent predictors of adverse pathological outcomes (e.g., ≥pT3 stage, nodal or distant metastasis), such analyses were not performed due to sample size limitations and the heterogeneity of cancer subtypes. Nonetheless, we recognize that differences in baseline patient characteristics—such as age, comorbidities, or tumor biology—could have acted as confounding factors. Ideally, a multivariable model including variables such as cohort (PREP vs. POSTP), age, comorbidity burden (e.g., Charlson Comorbidity Index), and tumor-specific risk factors (e.g., preoperative PSA, biopsy Gleason score) would clarify whether pandemic timing was an independent predictor of pathological stage progression.

### Bias and limitations

All surgeries were performed at the same institution by similar surgical teams, using standardized perioperative and pathological evaluation protocols to minimize institutional variability. However, potential selection bias must be acknowledged: during the pandemic, surgical triage policies may have led to prioritization of patients with more aggressive disease, potentially influencing the observed differences between groups. No formal matching procedure was applied between cohorts.

### Sensitivity Analyses

As an exploratory measure, the proportion of patients presenting with advanced pathological stage (defined as ≥pT3, N+, or M+) was assessed within each cancer group and compared across the two time periods. Survival data were not evaluated due to insufficient long-term follow-up.

## Results

### Prostate cancer

A total of 176 patients with PCa underwent RP—84 in the PREP group (47.7%) and 92 in the POSTP group (52.3%). The mean age was comparable between groups (66.2 ± 6.6 vs. 66.1 ± 6.4 years; p=0.91).

In the POSTP group, the preoperative PSA level was significantly higher (13.2 ± 16.2 ng/ml compared to 7.7 ± 4.5 ng/ml; p<0.001). The tumor percentage was also significantly higher in the POSTP group (17.0 ± 17.8 vs. 11.5 ± 13.0; p=0.019). The distribution of the Gleason score and International Society of Urological Pathology (ISUP) classification was similar between the two groups, with the most common pathology in both groups being 3 + 4 (ISUP 2).

The frequency of perineural invasion (PNI) was significantly higher in POSTP (88.0% vs. 73.8%; p=0.031), as was lymphovascular invasion (12.0% vs. 3.6%; p=0.018). Extraprostatic extension (EPE) was more common in POSTP (33.7% vs. 11.9%; p=0.006). Lymph node metastasis was detected only in POSTP (3.3%), but the overall number of cases was too small for statistical comparison.

**Figure 1:**
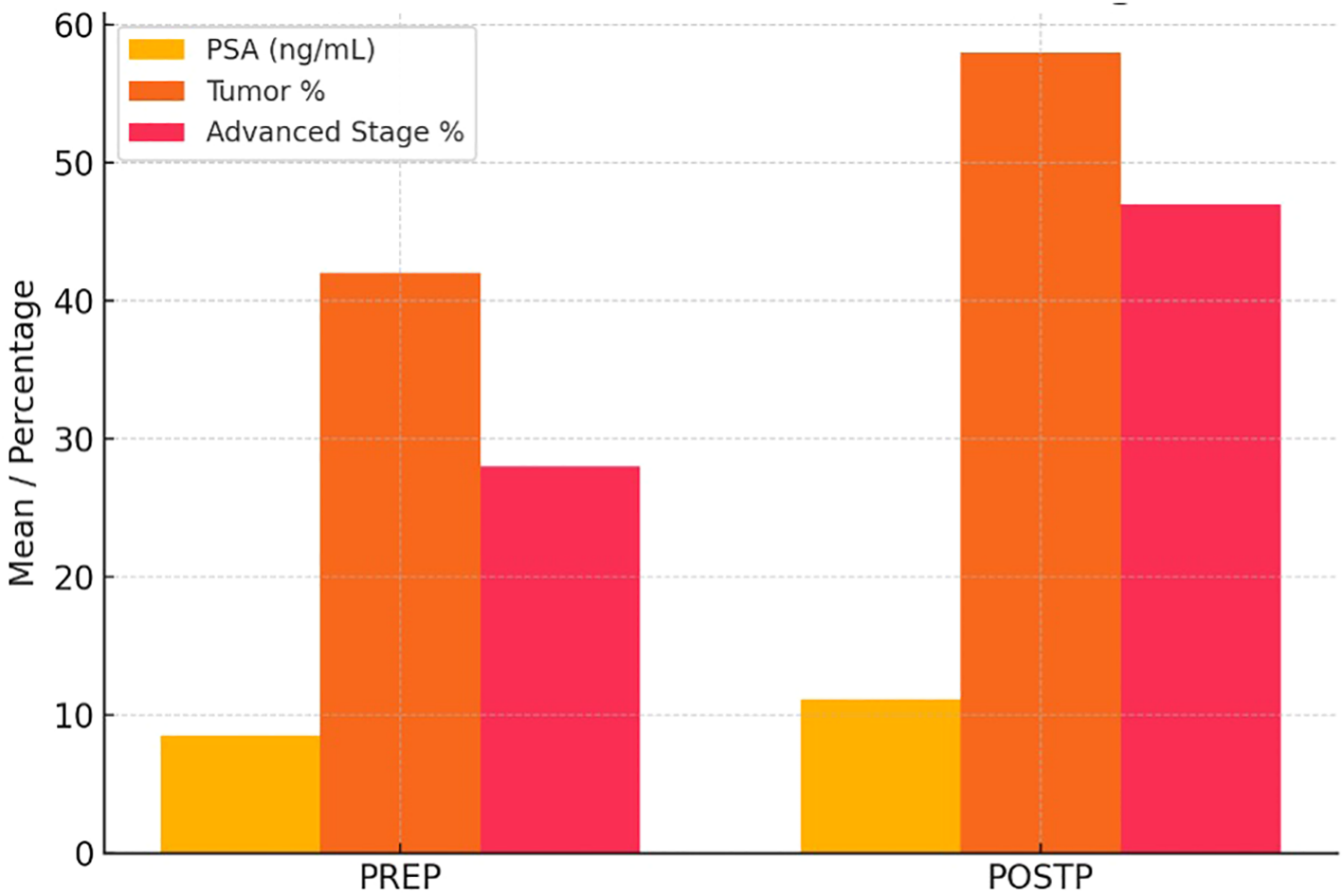
Pre- and Post-Pandemic PSA Levels, Tumor Percentage, and Stage Distribution in Prostate Cancer Patients. In the POSTP group, a statistically significant increase in PSA levels, tumor percentage, and the frequency of locally advanced stages was observed.

Pathological staging showed that stage 2C was the most frequent in both groups (52.4% PREP, 45.7% POSTP). One POSTP patient was diagnosed with stage 4 disease. These findings are detailed in **Table 1** and visually summarized in **Figure 1**.

**Table 1:** Comparative clinical and pathological characteristics in radical prostatectomy cases.

	PREP-RP (Mean ± SD)	POSTP-RP (Mean ± SD)	Mean-RP (Min-Max)	p
**Age**	66.2 ± 6.6	66.1 ± 6.4	66.1 (49.0-82.0)	0.95*
**Preoperative PSA (ng/ml)**	7.7 ± 4.5	13.2 ± 16.2	10.6 (0.3-118.0)	<0.001**
**Tumor Percentage**	11.5 ± 13.0	17.0 ± 17.8	14.4 (1.0-96.0)	0.02**

	PREP-RP n (%)	POSTP-RP n (%)	Mean-RP n (%)	p
Gleason Score				
3+3/6	25 (% 29.8)	18 (% 19.6)	43 (% 24.4)	
3+4/7	34 (% 40.5)	36 (% 39.1)	70 (% 39.8)	
3+5/8	1 (% 1.2)	6 (% 6.5)	7 (% 4.0)	
4+3/7	17 (% 20.2)	12 (% 13.0)	29 (% 16.5)	
4+4/8	3 (% 3.6)	6 (% 6.5)	9 (% 5.1)	
4+5/9	3 (% 3.6)	9 (% 9.8)	12 (% 6.8)	
5+3/8	0 (% 0.0)	1 (% 1.1)	1 (% 0.6)	
5+4/9	0 (% 0.0)	4 (% 4.3)	4 (% 2.3)	
5+5/10	1 (% 1.2)	0 (% 0.0)	1 (% 0.6)	
ISUP Grade				
ISUP-I	25 (% 29.8)	18 (% 19.6)	43 (% 24.4)	
ISUP-II	34 (% 40.5)	36 (% 39.1)	70 (% 39.8)	
ISUP-III	17 (% 20.2)	12 (% 13.0)	29 (% 16.5)	
ISUP-IV	4 (% 4.8)	13 (% 14.1)	17 (% 9.7)	
ISUP-V	4 (% 4.8)	13 (% 14.1)	17 (% 9.7)	
Tumor Location				
Right Lobe	11 (% 13.1)	14 (% 15.2)	25 (% 14.2)	
Left Lobe	16 (% 19.0)	11 (% 12.0)	27 (% 15.3)	
Apex	2 (% 2.4)	0 (% 0.0)	2 (% 1.1)	
Bilaterally	33 (% 39.3)	35 (% 38.0)	68 (% 38.6)	
Right Lobe-Apex	2 (% 2.4)	0 (% 0.0)	2 (% 1.1)	
Left Lobe-Apex	20 (% 23.8)	32 (% 34.8)	52 (% 29.5)	
Perineural Invasion				
No	22 (% 26.2)	17 (% 18.5)	39 (% 22.2)	0.28***
Yes	62 (% 73.8)	75 (% 81.5)	137 (% 77.8)	
Lymphovascular invasion				
No	81 (% 96.4)	81 (% 88.0)	162 (% 92.0)	0.05****
Yes	3 (% 3.6)	11 (% 12.0)	14 (% 8.0)	
Extra prostatic extension				
No	74 (% 88.1)	61 (% 66.3)	135 (% 76.7)	<0.001***
Yes	10 (% 11.9)	31 (% 33.7)	41 (% 23.3)	
Surgical Margin				
Negative	52 (% 61.9)	53 (% 57.6)	105 (% 59.7)	0.64***
Positive	32 (% 38.1)	39 (% 42.4)	71 (% 40.3)	
Lymph Node				
Negative	84 (% 100.0)	89 (% 96.7)	173 (% 98.3)	0.25****
Positive	0 (% 0.0)	3 (% 3.3)	3 (% 1.7)	
Pathological Stage				
Localized	73 (% 86.9)	63 (% 68.5)	136 (%77.3)	<0.001***
Locally advanced	11 (% 13.1)	29 (% 31.5)	40 (% 22.7)	
Pathological Stage (Summary)				
2A	29 (% 34.5)	21 (% 22.8)	50 (% 28.4)	
2C	44 (% 52.4)	42 (% 45.7)	86 (% 48.9)	
3A	4 (% 4.8)	16 (% 17.4)	20 (% 11.4)	
3B	7 (% 8.3)	12 (% 13.0)	19 (% 10.8)	
4	0 (% 0.0)	1 (% 1.1)	1 (% 0.6)	
HT				
No	45 (% 53.6)	46 (% 50.0)	91 (% 51.7)	
Yes	39 (% 46.4)	46 (% 50.0)	85 (% 48.3)	
DM				
No	59 (% 70.2)	66 (% 71.7)	125 (% 71.0)	
Yes	25 (% 29.8)	26 (% 28.3)	51 (% 29.0)	

PREP-RP: Radical Prostatectomy group before the COVID-19 pandemic.

POSTP-RP: Radical Prostatectomy group after the COVID-19 pandemic.

PSA, Prostate-Specific Antigen.

ISUP, International Society of Urological Pathology.

SD, Standard Deviation.

*Student’s t-testi **Mann–Whitney U testi ***Ki-kare (χ²) testi ****Fisher’s Exact Test.

### Bladder cancer

Seventy-eight patients with BCa underwent RC—37 in PREP and 41 in POSTP. The mean age was slightly higher in the POSTP group (68.3 ± 7.4 vs. 65.8 ± 13.9 years; p=0.27), and males predominated in both groups.

Preoperative hemoglobin was significantly lower in POSTP (10.4 ± 1.6 vs. 12.2 ± 2.0 g/dL; p<0.05). Creatinine levels (1.5 ± 0.9 vs. 1.2 ± 0.5 mg/dL) and tumor size (6.2 ± 3.2 vs. 5.4 ± 2.9 cm) were higher in POSTP, but these differences were not statistically significant.

Urothelial carcinoma was the predominant histological type in both cohorts (74.1% PREP, 62.7% POSTP). Tumor location was commonly multifocal in both groups (~50%).

The rate of positive surgical margins was higher in POSTP (29.4% vs. 14.8%), although the difference was not statistically significant (p=0.11). Lymph node metastases were more common in POSTP (69.6% vs. 48.1%; p=0.049), while the rate of distant metastases was similar (17.4% POSTP vs. 15.4% PREP; p=0.79).

Pathological staging showed a more varied distribution in POSTP: T3 was most frequent in PREP (37.0%), whereas both T2 and T4 occurred at 29.4% in POSTP. No significant difference in overall stage distribution was found. The POSTP group had more patients with hypertension (62.7% vs. 48.1%) and fewer with diabetes (27.5% vs. 37.0%). **Table 2** presents a comparative evaluation of the PREP and POSTP RC findings.

**Table 2:** Clinical and pathological comparison of bladder cancer patients undergoing radical cystectomy.

	PREP-RC (Mean ± SD)	POSTP-RC (Mean ± SD)	Mean-RC (Min-Max)	p
**Age**	65.8 ± 13.9	68.3 ± 7.4		0.71*
**Preoperative HGB (g/dL)**	12.2 ± 2.0	10.4 ± 1.6		<0.001*
**Preoperative Creatinine (mg/dL)**	1.2 ± 0.5	1.5 ± 0.9		0.18**
**Tumor Size (cm)**	5.4 ± 2.9	6.2 ± 3.2		0.26**

	PREP-RC n (%)	POSTP-RC n (%)	Mean-RC n (%)	p
Gender				
Female	3 (% 11.1)	7 (% 13.7)	10 (%12.8)	0.52***
Male	24 (% 88.9)	44 (% 86.3)	68 (% 87.2)	
Pathological Type				
Urothelial carcinoma	20 (% 74.1)	32 (% 62.7)	52 (% 66.7)	0.45***
Variant pathology	7 (% 25.9)	19 (% 37.3)	26 (% 33.3)	
Location in the Bladder				
Left side wall	4 (% 14.8)	7 (% 13.7)	11(% 14.1)	
Right side wall	5 (% 18.5)	9 (% 17.6)	14 (% 17.9)	
Base	2 (% 7.4)	6 (% 11.8)	8 (% 10.3)	
Opposite wall	0 (% 0.0)	4 (% 7.8)	4 (% 5.1)	
Dome	1 (% 3.7)	2 (% 3.9)	3 (% 3.8)	
Multiple wall involvement	15 (% 55.6)	23 (% 45.2)	38(% 48.7)	
Concomitant PCa				
No	24 (% 88.9)	43 (% 84.3)	67 (% 85.9)	0.74***
Yes	3 (% 11.1)	8 (% 15.7)	11 (% 14.1)	
Surgical Margin				
Negative	23 (% 85.2)	36 (% 70.6)	59 (% 75.6)	0.18****
Positive	4 (% 14.8)	15 (% 29.4)	19 (% 24.4)	
Metastasis				
No	14 (% 51.9)	28 (% 54.9)	42 (% 53.8)	0.82***
Yes	13 (% 48.1)	23 (% 45.1)	36 (% 46.2)	
Metastasis Location				
Retroperitoneal	9 (% 69.2)	16 (% 69.6)	25 (% 69.4)	
Distant metastasis	2 (% 15.4)	4 (% 17.4)	6 (% 16.7)	
Both conditions	2 (% 15.4)	3 (% 13.0)	5 (% 13.9)	
Pathological Stage				
T1	6 (% 22.2)	7 (% 13.7)	13 (% 16.7)	0.28***
T2	8 (% 29.6)	15 (% 29.4)	23 (% 29.5)	
T3	10 (% 37.0)	14 (% 27.5)	24 (% 30.8)	
T4	3 (% 11.1)	15 (% 29.4)	18 (% 23.1)	
HT				
No	14 (% 51.9)	19 (% 37.3)	33 (% 42.3)	0.24***
Yes	13 (% 48.1)	32 (% 62.7)	45 (% 57.7)	
DM				
No	17 (% 63.0)	37 (% 72.5)	54 (% 69.2)	0.44***
Yes	10 (% 37.0)	14 (% 27.5)	24 (%30.8)	

PREP-RC: Radical Cystectomy group before the COVID-19 pandemic.

POSTP-RC: Radical Cystectomy group after the COVID-19 pandemic.

HGB, Hemoglobin.

SD, Standard Deviation.

HT, Hypertension.

DM, Diabetes Mellitus.

*Student’s t-testi **Mann–Whitney U ***Ki-kare (χ²) testi ****Fisher’s Exact Test.

### Renal cell carcinoma

Seventy-eight patients with renal cell carcinoma (RCC) underwent RN—39 in each cohort. Sex distribution was similar (PREP: 68.3% male; POSTP: 61.0% male). Median age was 68 years in both groups.

Hemoglobin levels were significantly lower in POSTP (11.7 g/dL vs. 12.9 g/dL; p<0.001), and creatinine was higher (1.1 mg/dL vs. 0.9 mg/dL; p=0.043). Tumor size was significantly larger in POSTP (7.4 cm vs. 6.0 cm; p=0.01).

Clear cell carcinoma remained the predominant subtype (68.3% PREP, 55.9% POSTP). Pathologically, advanced stage (≥pT3) tumors were more frequent in POSTP (T3: 35.6% vs. T1: 61.0% in PREP), although stage differences were not statistically significant.

Metastases were present in 20.3% of POSTP patients and 9.8% in PREP (p=0.09). Lymph node metastasis was also more common in POSTP (22.0% vs. 9.8%). Surgical margins were rarely positive in either group (2.4% PREP, 3.4% POSTP).

**Figure 2:**
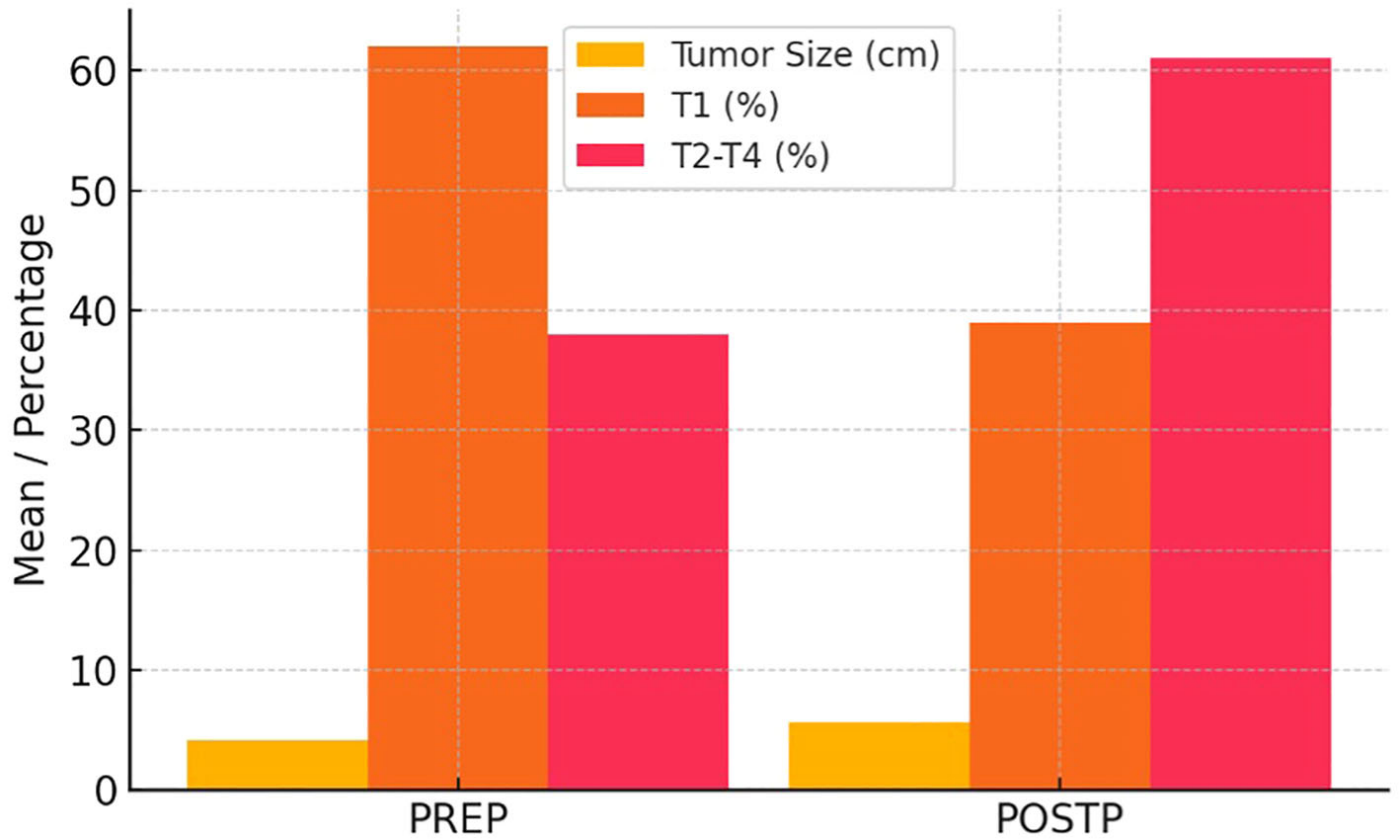
Tumor size and pathological stage distribution in RCC across PREP and POSTP.

**Table 3** shows a comparative evaluation of PREP and POSTP RCC findings. **Figure 2** illustrates the increase in tumor size and the significant rise in stages beyond T1 in the POSTP group.

**Table 3:** Comparison of clinical and pathological characteristics in radical nephrectomy.

	PREP-RN	POSTP-RN	Mean-RN	p
	(Mean ± SD)	(Mean ± SD)	(Min-Max)	
Age	68	68		0.98*
Preoperative HGB (g/dL)	12.9	11.7		<0.001*
Preoperative Creatinine (mg/dL)	0.9	1.1		<0.001**
Tumor Size (cm)	6	7.4		0.01**
	n (%)	n (%)	n (%)	
Gender				
Female	13 (% 31.7)	23 (% 39.0)	36 (%36.0)	0.53***
Male	28 (% 68.3)	36 (% 61.0)	64 (%64.0)	
Pathological Type				
Clear cell	28 (% 68.3)	33 (% 55.9)	61 (%61.0)	0.40***
Papillary	8 (% 19.5)	11 (% 18.6)	26 (%26.0)	
Chromophobe	2 (% 4.9)	5 (% 8.5)	7 (%7.0)	
Others	3 (% 7.3)	10 (% 16.9)	13 (%13.0)	
Tumor Location				
Upper pole	9 (% 22.0)	19 (% 32.2)	28 (% 28.0)	
Upper and middle pole	6 (% 14.6)	6 (% 10.2)	12 (% 12.0)	
Middle pole	10 (% 24.4)	10 (% 16.9)	20 (% 20.0)	
Middle and lower pole	6 (% 14.6)	14 (% 23.7)	20 (% 20.0)	
Lower pole	9 (% 22.0)	10 (% 16.9)	19 (% 19.0)	
Surgical Margin				
Negative	40 (% 97.6)	57 (% 96.6)	97 (%97.0)	1.0****
Positive	1 (%2.4)	2 (% 3.4)	3 (%3.0)	
**Metastasis**				
No	37 (% 90.2)	47 (% 79.7)	84 (% 84.0)	0.18***
Yes	4 (% 9.8)	12 (% 20.3)	16 (%16.0)	
Retroperitoneal Lymph Node Metastasis				
No	36 (% 87.8)	46 (% 78.0)	82 (%82.0)	0.29***
Yes	5 (% 12.2)	13 (% 22.0)	18 (%18.0)	
Pathological Stage				
T1	25 (% 61.0)	24 (% 40.7)	49 (% 49.0)	0.09***
T2	9 (% 22.0)	12 (% 20.3)	21 (% 21.0)	
T3	6 (% 14.6)	21 (% 35.6)	27 (%27.0)	
T4	1 (% 2.4)	2 (% 3.4)	3 (%3.0)	
HT				
No	18 (% 43.9)	26 (% 44.1)	44 (% 44.0)	
Yes	23 (% 56.1)	33 (% 55.9)	56 (% 56.0)	
DM				
No	29 (% 70.7)	50 (% 84.7)	79 (%79.0)	
Yes	12 (% 29.3)	9 (% 15.3)	21 (%21)	

PREP-RN: Radical Nephrectomy group before the COVID-19 pandemic.

POSTP-RN: Radical Nephrectomy group after the COVID-19 pandemic.

HGB, Hemoglobin.

SD, Standard Deviation.

HT, Hypertension.

DM, Diabetes Mellitus.

*Student’s t-testi **Mann–Whitney U testi ***Ki-kare (χ²) testi ****Fisher’s Exact Test.

### Testicular cancer

Thirty-six patients with TCa underwent RO—11 in PREP (30.6%) and 25 in POSTP (69.4%). The median age was similar (27.0 vs. 31.0 years; p=0.48).

Tumor markers (AFP, β-hCG, LDH) and tumor size (5.0 vs. 4.2 cm) were comparable between groups, with no statistically significant differences (all p>0.5).

Mixed germ cell tumor was the most frequent histologic subtype in both groups (PREP: 54.5%, POSTP: 48.0%). LVI was more common in POSTP (44.0% vs. 18.2%), and rete testis invasion was observed in 11 POSTP patients and 3 PREP patients, though these findings were not statistically significant.

Positive surgical margins were only observed in PREP (18.2%). Most cases in both groups were pathologic stage T1 (PREP: 72.2%, POSTP: 60.0%). Retroperitoneal lymph node involvement was more frequent in PREP (63.6% vs. 44.0%), while distant metastasis rates were similar (~16–18%). A comparative evaluation of PREP and POSTP RO findings is presented in **Table 4**.

**Table 4:** Comparison of clinical and pathological features in radical orchiectomy cases.

	PREP-O	POSTP-O	Mean-RO	p
	(Mean ± SD)	(Mean ± SD)	(Min-Max)	
Age	27	31		0.25*
Preoperative AFP (ng/mL)	105	9.1		0.59**
Preoperative β-HCG (IU/L)	2.9	2.5		0.90**
Preoperative LDH (IU/L)	180	202		0.74**
Tumor Size (cm)	5	4.2		0.87**
	n (%)	n (%)	n (%)	
Pathological Type				
Embryonal carcinoma	1 (% 9.1)	2 (% 8.0)	3(% 8.4)	
Leiomyosarcoma	0 (% 0.0)	1(% 4.0)	1 (% 2.8)	
Malignant lymphoma	0 (% 0.0)	1 (% 4.0)	1 (% 2.8)	
Mature cystic teratoma	0 (% 0.0)	1 (% 4.0)	1 (% 2.8)	
Mixed germ cell tumor	6 (% 54.5)	12 (% 48.0)	18 (% 50.0)	
Regressed germ cell tumor (burn out)	1 (%9.1)	0 (% 0.0)	1 (% 2.8)	
Seminoma	3 (% 27.3)	8 (% 32.0)	11 (% 30.6)	
Rete Testis Involvement				
No	8 (% 72.7)	14 (% 56.0)	22 (% 61.1)	0.57***
Yes	3 (% 27.3)	11 (% 44.0)	14 (% 38.9)	
Spermatic Cord Invasion				
No	10 (% 90.9)	23 (% 92.0)	33 (% 91.7)	1.00****
Yes	1 (% 9.1)	2 (% 8.0)	3 (% 8.3)	
Lymphatic Invasion				
No	9 (% 81.8)	14 (% 56.0)	23 (% 63.9)	0.26***
Yes	2 (% 18.2)	11 (% 44.0)	13 (% 36.1)	
Surgical Margin				
Negative	9 (% 81.8)	24 (% 96.0)	33 (% 91.7)	0.22****
Positive	2 (% 18.2)	1 (% 4.0)	3 (% 8.3)	
Metastasis				
No	9 (% 81.8)	21 (% 84.0)	30 (% 83.3)	1.00****
Yes	2 (% 18.2)	4 (% 16.0)	6 (% 16.7)	
Metastasis Location				
Pulmonary	2 (% 100.0)	3 (% 75.0)	5 (% 83.3)	1.00****
Extrapulmonary	0 (% 0.0)	1 (% 25.0)	1 (% 16.7)	
Retroperitoneal Lymph Node Metastasis				
No	7 (% 63.6)	11 (% 44.0)	18 (% 50.0)	0.47***
Yes	4 (% 36.4)	14 (% 56.0)	18 (% 50.0)	
Pathological Stage				
T1	8 (% 72.7)	15 (% 60.0)	23 (% 63.9)	0.54***
T2	2 (% 18.2)	7 (% 28.0)	9 (% 25.0)	
T3	1 (% 9.1)	3 (% 12.0)	4 (% 11.1)	
HT				
No	10 (% 90.9)	20 (% 80.0)	30 (% 83.3)	0.64***
Yes	1 (% 9.1)	5 (% 20.0)	6 (% 16.7)	
DM				
No	11 (% 100.0)	23 (% 92.0)	34 (% 94.4)	1.00****
Yes	0 (% 0.0)	2 (% 8.0)	2 (% 5.6)	

PREP-O: Radical Orchiectomy group before the COVID-19 pandemic.

POSTP-O: Radical Orchiectomy group after the COVID-19 pandemic.

AFP, Alpha-Fetoprotein.

β-HCG, Beta-Human Chorionic Gonadotropin.

LDH, Lactate Dehydrogenase.

SD, Standard Deviation.

HT, Hypertension.

DM, Diabetes Mellitus.

*Student’s t-testi **Mann–Whitney U testi ***Ki-kare (χ²) testi ****Fisher’s Exact Test.

**Figure 3:**
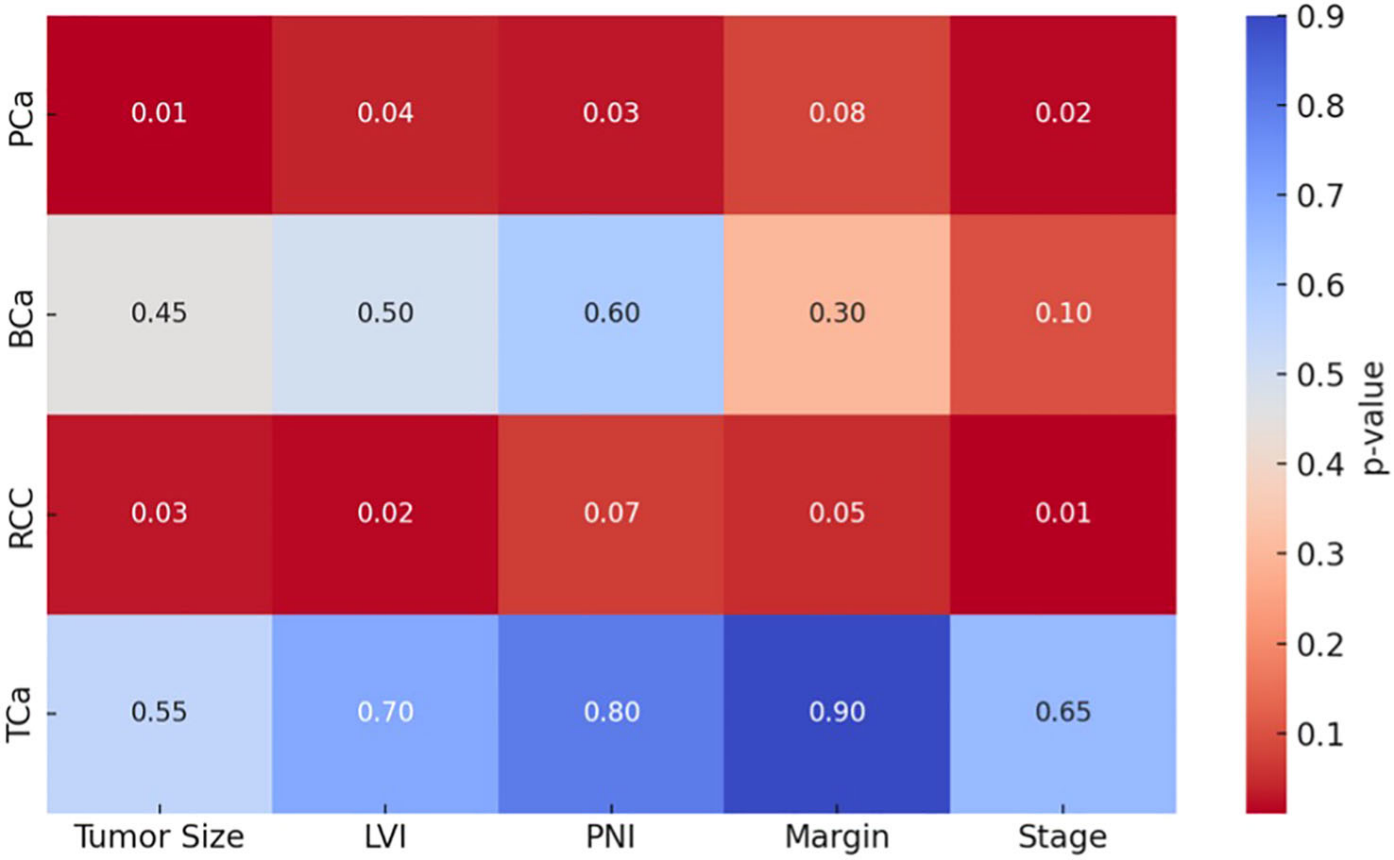
Heatmap visualization of significant pathological parameter shifts across tumor types (darker cells = p<0.05). Each row represents a different cancer type (PCa: Prostate, BCa: Bladder, RCC: Kidney, TCa: Testis), and each column represents the histopathological parameter being compared. The color intensity indicates the statistical significance (p-value).

**Figure 3** illustrates a heatmap of p-values comparing key pathological parameters (tumor size, LVI, PNI, surgical margin, and pathological stage) between PREP and POSTP periods across four urological cancers. Statistically significant differences (p < 0.05) were predominantly observed in the PCa and renal cell carcinoma (RCC) groups, particularly for tumor size, lymphovascular invasion (LVI), perineural invasion (PNI), and pathological stage. In contrast, bladder cancer (BCa) and testicular cancer (TCa) showed no statistically significant changes (p > 0.1 for all comparisons), as reflected by lighter-colored cells in the heatmap.

## Discussion

Our comparative analysis of uro-oncologic surgeries performed before and after the COVID-19 pandemic revealed several statistically significant shifts toward more aggressive disease features in the post-pandemic cohort. Among PCa patients, the post-pandemic group exhibited markedly higher median PSA levels, increased tumor involvement, and a significantly greater prevalence of PNI, LVI, and EPE. In the BCa cohort, lymph node metastasis was significantly more common in the post-pandemic group. Similarly, in renal cell carcinoma, tumor size was significantly larger, hemoglobin was lower post-pandemic. While TCa did not show statistically significant differences, there was a noticeable increase in the frequency of lymphovascular and rete testis invasion after the pandemic. These findings collectively suggest a pattern of stage migration and delayed presentation across multiple urologic malignancies in the aftermath of pandemic-related healthcare disruptions.

### Prostate cancer

In our series, RP specimens from the POSTP displayed significantly more aggressive features than those from the pre-pandemic period. Median PSA at surgery rose markedly (13.2 vs. 7.7 ng/mL; *p*<0.001), and the mean tumor involvement of the gland increased (17.0% vs. 11.5%; *p*=0.019). Adverse pathology was more frequent post-pandemic: PNI occurred in 88% of cases versus 73.8% (p=0.031), LVI in 12.0% vs. 3.6% (p=0.018), and EPE in 33.7% vs. 11.9% (p=0.006). In contrast, the distribution of ISUP (Gleason) grade groups did not differ significantly between eras. Lymph node metastases were seen only in the post-pandemic cohort (occurring in a small number of patients), precluding formal statistical comparison for that outcome.

These findings likely reflect shifts in case selection rather than an intrinsic change in tumor biology. During the pandemic, many centers deferred elective RP for low-risk disease and reserved surgical capacity for higher-risk cancers (8). Detti et al. explicitly noted that RP for indolent tumors could be postponed until after the COVID-19 surge. Concurrently, routine PSA screening and outpatient visits declined, so fewer early-stage cancers were diagnosed. An Italian registry study found roughly 20–21% fewer early-stage PCa diagnosed in 2020 than expected, with a relative increase in advanced or metastatic presentations (10). Taken together, these shifts mean that the cohort of men operated during the pandemic was likely enriched for adverse pathology (high PSA, larger tumors, invasion) while many low-risk patients were underrepresented. Indeed, Andrade et al. reported no pandemic-associated change in RP pathological stage or grade at their center underscoring how differences in referral patterns and practice can influence results (11).

It is difficult to establish a direct causal link between the COVID-19 pandemic and the emergence of more aggressive cancer features. Notably, the number of patients with lymph node-positive disease was very limited, rendering any observed differences in nodal status unreliable. Therefore, although we observed a trend toward higher-risk PCa characteristics in the POSTP, this is more likely to reflect a shift in case mix—such as the triage of sicker patients, reduced screening activities, and delays in referrals—rather than a direct biological impact of the pandemic on tumor behavior.

### Bladder cancer

In our cohort of patients undergoing RC, those treated during the POSTP period showed a notably higher rate of lymph node metastases at surgery (69.6% vs 48.1%, p=0.049) and an increased, though not statistically significant, rate of positive surgical margins (29.4% vs 14.8%, p=0.11). Preoperative hemoglobin was significantly lower in the post-pandemic group, and both tumor size and serum creatinine tended to be higher, albeit not reaching significance. Histopathology remained overwhelmingly urothelial carcinoma in both groups, with multifocal tumors predominating; distant metastases at RC time were similar pre- and post-pandemic.

The marked increase in nodal disease in the COVID-era cohort is consistent with other reports: for example, Oderda et al. found that RC specimens from 2020 had significantly more lymph node involvement and extravesical extension than in 2019 (12). Likewise, Anderson et al. observed fewer BCa cases treated during COVID with a higher proportion of invasive or high-grade tumors, suggesting that patients tended to present later in the course of disease after the pandemic began (13). These shifts likely reflect temporal associations rather than a direct causal effect of the pandemic. Delays in diagnosis or referrals during COVID could select for more advanced tumors at the time of surgery. For instance, multi-institutional surveys reported that a non-negligible fraction of centers temporarily delayed RC (approximately 10–19% of sites at pandemic peaks) even as most urologic surgeries were maintained (14), implying heterogeneous triage and referral patterns. In interpreting our data, it is important to note that only patients undergoing RC were included – those managed with TURBT alone were excluded – which may bias the cohort toward more aggressive cases and limit generalizability.

Therefore, while our findings highlight a concerning trend toward more advanced BCa (higher nodal burden and margin positivity) in the post-pandemic period, they represent associations in timing and presentation, not proof of causality. These results underscore the need for continued vigilance in timely cancer diagnosis and treatment allocation and are in line with emerging literature on pandemic-related stage migration in urothelial carcinoma.

### Renal cell carcinoma

Our analysis of 78 RCC patients undergoing RN revealed notably more advanced disease in the post-pandemic group. Specifically, patients treated after the onset of COVID-19 had significantly larger tumors (mean 7.4 cm vs 6.0 cm) and more frequent lymph node metastases (22.0% vs 9.8%), as well as lower preoperative hemoglobin and higher creatinine. Although the higher proportion of pT3 tumors in the post-pandemic cohort did not reach statistical significance, the trend toward more advanced pathological stage is concordant with recent reports. These findings mirror broader trends in oncology: multiple studies have documented a sharp decline in new cancer diagnoses during the pandemic, especially for early-stage and low-risk tumors (15, 16). Yildirim et al. found a 15–25% drop in new RCC diagnoses during the first COVID wave – overwhelmingly affecting small (T1a/T1b) tumors and elderly patient (15).

National data likewise showed that kidney cancer exhibited one of the largest relative declines in incidence and a shift away from early-stage presentation in 2020 (17). In sum, our post-pandemic cohort appears enriched for patients with higher disease burden at presentation, a pattern in keeping with these observed reductions in early RCC detection.

Concomitant literature supports a pandemic-associated “stage migration” in RCC. In a single-center analysis of 184 patients, Gupta et al. reported that mean tumor size was significantly greater during the COVID era (7.10 cm vs 5.84 cm, p=0.017) (18). That study also observed a higher number of metastatic cases in the pandemic cohort (7 vs 1, p=0.042) (18). Similarly, Janes et al. found that in the year following the first COVID waves, the proportion of pT3 RCC rose from ~35–39% pre-pandemic to 50% post-pandemic (p=0.003), together with significantly longer surgical wait times (19). These authors concluded that their findings indicated a “clinically significant stage migration,” likely driven by diagnostic and treatment delays. Our observation of larger tumors and more nodal involvement after the pandemic is fully consistent with these reports. Lymph node metastasis in RCC is a known adverse prognostic factor, and its increased frequency in our post-COVID cohort (22% vs 9.8%) suggests that many patients presented with more aggressive disease.

Multiple pandemic-related factors likely contributed to the observed stage progression in renal cell carcinoma (RCC). First, diagnostic delays due to lockdowns and patient hesitancy significantly reduced early tumor detection. Since approximately 70% of RCC cases are identified incidentally via imaging, the suspension of elective radiologic evaluations led to a notable decline in the diagnosis of small, asymptomatic tumors (20). As a result, patients presenting after restrictions were lifted more often had larger or symptomatic tumors (15). Second, surgical prioritization guidelines classified small renal masses (cT1a) as deferrable up to six months, while larger or symptomatic tumors were treated urgently (21). This triaging likely allowed some early-stage tumors to progress prior to intervention, contributing to the higher proportion of ≥pT3 cases in our POSTP cohort. Third, prolonged surgical wait times added to treatment delays. Janes et al. reported significantly longer intervals from diagnosis to RN during the pandemic, which may increase the risk of tumor progression and nodal spread (19). In our cohort, lymph node metastasis was more than twice as frequent post-pandemic (22.0% vs. 9.8%).

Taken together, these mechanisms align with broader epidemiological patterns reported during COVID-19 and support the interpretation that pandemic-associated disruptions led to delayed RCC presentation and more aggressive pathological features. Continued surveillance and timely intervention will be crucial to prevent further stage migration.

### Testicular cancer

In this study we found that basic demographic and tumor characteristics (age, tumor size, and serum markers) were essentially unchanged between the PREP and POSTP cohorts. Most tumors in both groups were pathologically Stage T1, consistent with typical TCa series (approximately 75–80% of seminoma and 55–64% of non‐seminoma present as clinical Stage I). There were no significant differences in age or marker levels, and median tumor sizes overlapped in the two cohorts, suggesting that pandemic-related disruptions did not delay diagnosis in a detectable way for the majority of patients.

Notably, the POSTP cohort showed a higher proportion of cases with lymphovascular invasion (LVI) and rete testis invasion (RTI). Although these differences were not statistically significant (likely due to our modest sample size), they are clinically intriguing. Both LVI and RTI are well‐recognized adverse prognostic features in testicular germ cell tumors. For nonseminomatous tumors, LVI is the strongest validated risk factor for relapse: historically, roughly 50% of patients with LVI-positive Stage I disease will relapse versus only about 15% if LVI is absent (22, 23). In seminoma, invasion of the rete testis or testicular hilum has been associated with increased relapse risk. Indeed, prior studies have identified tumor size and RTI as predictors of relapse in clinical Stage I seminoma, and recent large cohorts confirm that hilum invasion (rete testis and hilar soft tissue) and LVI independently predict relapse risk (24). In our data, the trend toward more frequent LVI and RTI in POSTP cases—if borne out in larger series—could portend a subtle shift toward higher-risk pathology. Even modest elevations in these factors might justify closer surveillance or more aggressive adjuvant therapy.

By contrast, the greater retroperitoneal nodal involvement in PREP cases was unexpected, and its significance is unclear. It may reflect random variation or differences in referral patterns rather than a true clinical shift. Importantly, none of the observed trends (higher LVI/RTI or nodal involvement) reached statistical significance. This likely reflects limited power with our sample size, and suggests caution in over-interpreting the patterns. Nonetheless, we note that even non-significant changes in established risk factors could have implications: TCa relapse rates depend strongly on pathology. For example, it is known that patients with CS I nonseminoma and LVI have much higher relapse rates and current surveillance protocols often stratify follow-up intensity based on such risk features (25).

Although the literature on TCa during the COVID-19 pandemic is limited, our findings suggest that its detection and initial management remained relatively unaffected. Unlike malignancies relying on screening, testicular tumors typically present with symptoms such as a palpable mass, prompting timely medical attention. Pandemic-related disruptions led to declines in cancer diagnoses overall, but stage at diagnosis in our cohort remained early, supporting the notion that TCa was prioritized (26). Urological guidelines indeed recommended urgent management for suspected testicular tumors (27). While lymphovascular and rete testis invasion appeared more frequent in the POSTP group, these differences were not statistically significant and may reflect sample size limitations. Nevertheless, these known relapse risk factors warrant close follow-up. Larger studies are needed to clarify whether the pandemic subtly altered the risk profile or outcomes in this population.

This study has several limitations that warrant consideration. First, its retrospective and single-center design inherently limits causal inference and generalizability. Second, only patients who underwent definitive surgical treatment were included; those with inoperable, metastatic, or unresectable disease were excluded, introducing a potential selection bias. This may have led to underrepresentation of the most aggressive cases during the pandemic. Third, the absence of long-term oncologic follow-up precludes conclusions regarding survival outcomes or relapse. Fourth, diagnostic and referral patterns may have shifted during the pandemic, resulting in case-mix variation rather than true biological change—a possibility supported by prior literature. Additionally, the relatively small sample size in some subgroups, particularly TCa, may have limited the statistical power to detect significant differences. Lastly, unmeasured confounders such as regional health policy changes, patient socioeconomic status, and variable institutional triage practices during COVID-19 may have further influenced our findings.

## Conclusion

The COVID-19 pandemic has inflicted deep wounds not only in the management of infectious diseases but also in the diagnosis, monitoring, and treatment of chronic and oncological conditions. In our study, pathological findings indicated that the pandemic negatively impacted access to surgery and continuity of diagnosis in PCa and RCC, leading to stage progression. Although changes in some parameters were observed in BC and TCa, these findings did not reach statistical significance.

Our data vividly highlight the fragility of healthcare systems under crisis conditions and the profound consequences of delays in disease staging. These findings underscore the importance of rethinking oncological management in emergencies, not only in the context of pandemics but also in the face of future global crises such as wars, migration, and natural disasters. Our study offers essential insights into how oncological priorities should be established under extraordinary conditions, with implications extending far beyond the pandemic.

## Data Availability

The raw data supporting the conclusions of this article will be made available by the authors, without undue reservation.
